# Halotropism: Phytohormonal Aspects and Potential Applications

**DOI:** 10.3389/fpls.2020.571025

**Published:** 2020-09-17

**Authors:** Ágnes Szepesi

**Affiliations:** Department of Plant Biology, Institute of Biology, Faculty of Science and Informatics, University of Szeged, Szeged, Hungary

**Keywords:** halotropism, salinity, sodium, root system architecture, phytohormones

## Abstract

Halotropism is a sodium specific tropic movement of roots in order to obtain the optimal salt concentration for proper growth and development. Numerous results suggest that halotropic events are under the control and regulation of complex plant hormone pathway. This minireview collects some recent evidences about sodium sensing during halotropism and the hormonal regulation of halotropic responses in glycophytes. The precise hormonal mechanisms by which halophytes plant roots perceive salt stress and translate this perception into adaptive, directional growth forward increased salt concentrations are not well understood. This minireview aims to gather recently deciphered information about halotropism focusing potential hormonal aspects both in glycophytes and halophytes. Advances in our understanding of halotropic responses in different plant species could help these plants to be used for sustainable agriculture and other future applications.

## Introduction

### Halotropism

Halotropism a relatively new discovered type of tropism in plants, allowing them to escape from high salt by bending. Plant roots have ability to move from high salinity to avoid growth retardation or cell death. However, recently new findings show that some halophyte plant species require to obtain optimal salt concentration for their optimal growth (Shelef et al., 2016). Continuous sensing and searching for optimal salt concentration in soil or water needs different mechanisms. Halotropism is a sodium specific tropic movement of roots (Galvan-Ampudia et al., 2013) and not overlap with hydrotropism (Feng et al., 2016). The perception of sodium is supposed to be in the root as this is the first organ meet with salt containing soil. Root system architecture (RSA) can be remodeled during salinity (Koevoets et al., 2016). Recently, the genetic components of root architecture remodeling after salt stress were described by Julkowska et al. (2017).

**Figure 1:**
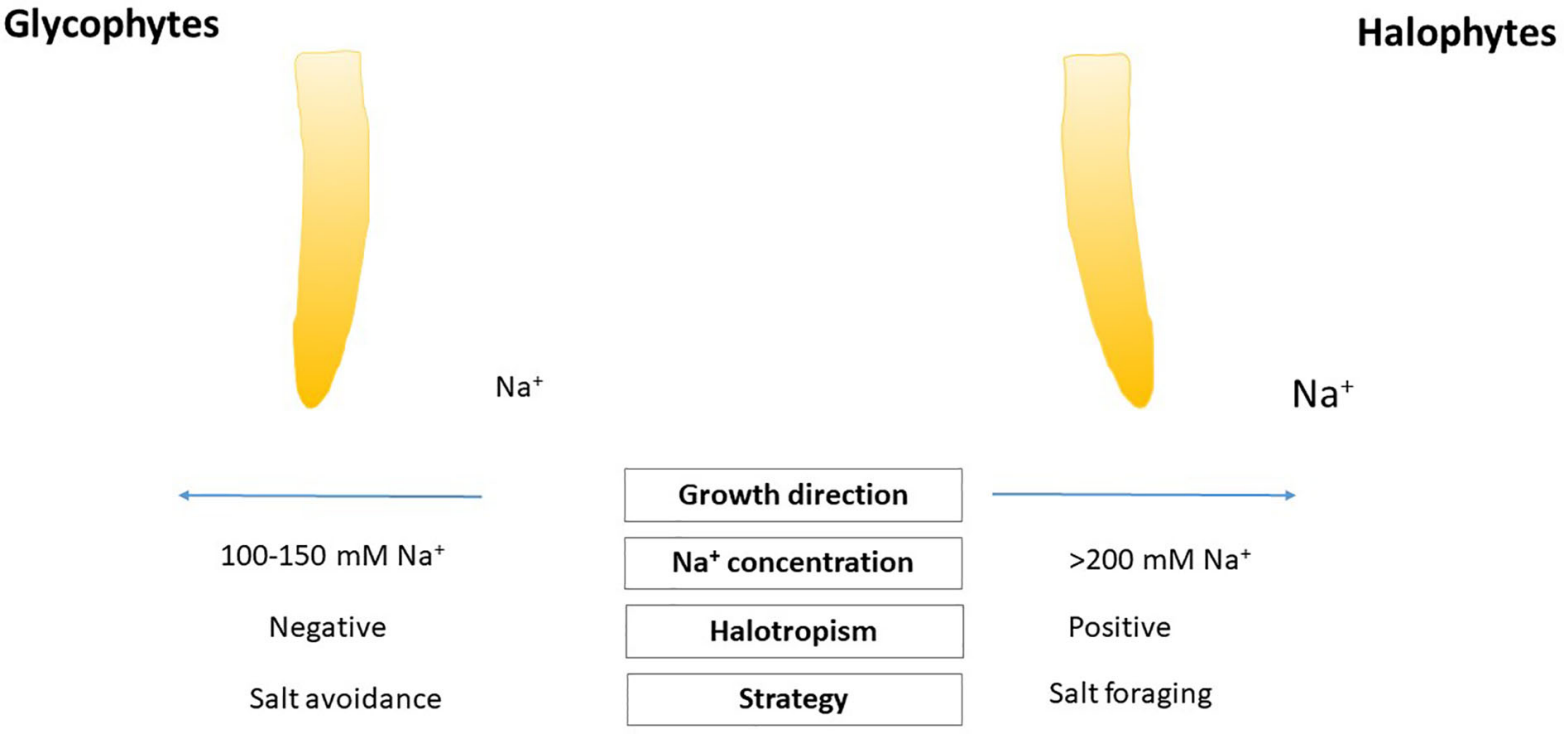
Model summarizing two types of halotropism in glycophytes and halophytes.

Plants differently respond to higher salt concentrations in the soil (Lamers et al., 2020). For salt sensitive glycophyte plants, higher salt concentrations can be harmful for their normal development and growth (Yang and Guo, 2018; van Zelm et al., 2020), while some halophytes developed some efficient strategies to survive high salinity in the soil and maintain salt concentration for their optimal growth (Fan, 2020). There is a hypothesis that glycophyte plant species show negative halotropism (Li and Zhang, 2008; Galvan-Ampudia et al., 2013) orientating their roots from supraoptimal salt concentration in the soil, however, some halophytes depending from their halophyte features can respond by positive halotropism for reaching optimal salt concentration to their normal development (Shelef et al., 2016). Positive halotropic movements discovered in some halophytes, in *Bassia indica* or *Limonium bicolor* (Sun et al., 2008; Shelef et al., 2016; Leng et al., 2019). It should be keep in mind features of halotropism focusing the different salt exclusion strategies of halophytes remain to be elucidated. There are very scarce studies on halotropic movements of other halophytes, euhalophytes or recretohalophytes. It will be of significant interest to look on the effect of non-homogenous salt soil conditions and understand the features of positive halotropism, as suboptimal soil conditions (e.g., poor nutrient supply) can affect salt driven mechanisms (Shelef et al., 2016). During halotropism, the gravitropic responses of roots should be repressed, so this mechanism may help roots to modify and fine tune their movement to optimal growth and survive high salt conditions (Galvan-Ampudia et al., 2013) (**Figure 1**). NASA plant life based solutions root tropisms are important in space conditions (Muthert et al., 2020).

## Sensing and Perception of Sodium During Halotropism

In spite of sodium sensor is unknown (Rosquete and Kleine-Vehn, 2013), there are some evidence that plants can selectively perceive and allocate the cation Na^+^ during halotropism (Dietrich et al., 2017; Deolu-Ajayi et al., 2019). It is likely that for halotropic reaction the action region of root could be in the elongation zone (Yokawa et al., 2014; van den Berg et al., 2016). The proper level of sodium which can trigger halotropic movements are different in halophytes (**Figure 1**). The exact sodium concentration range of halotropism is different in glycophytes and halophytes. In case of glycophyte *Arabidopsis thaliana*, 50–100 mM NaCl treatment provoked remarkable root bending as a feature of halotropism (Sun et al., 2008), and showed negative halotropism after 150 mM NaCl to avoid salt injury (Galvan-Ampudia et al., 2013). Halophytes may tune their halotropism at higher sodium level, but these investigations are needed to be investigated multiple and combined approaches. Some evidence suggests that relative high 200 mM NaCl could induce halotropic bending in some halophytes. SOS (salt overly sensitive) signal pathway plays a crucial role in halotropism. SOS1 is a Na^+^/H^+^-antiporter (Shi et al., 2000). The unknown sodium-specific sensor responsible for halotropic response is expected to sense the intracellular Na^+^ concentration, because the *sos1* mutant, which contains higher intracellular Na^+^ (Shi et al., 2002) showed an enhanced halotropic response (Galvan-Ampudia et al., 2013). New findings suggest that 14-3-3 proteins and other candidates could affect the transport activity of SOS1 forming protein-protein interactions with its cytosolic C-terminal end, enhancing our knowledge of this protein involved in salt avoidance mechanisms of roots (Duscha et al., 2020). Comparing the transcript levels of *SOS1* in *Eutrema* (*Thellungiella*) species, the halophyte relatives of *Arabidopsis* revealed that the basal and salt stressed induced expression of SOS1 was higher compared to the glycophytes (Oh et al., 2009), suggesting that different magnitude of Na^+^ sensing and regulation of halotropic events in halophytes.

## Components of Cellular and Physiological Features of Root Halotropism

Phospholipid signaling is also critical in inducing halotropic movement of roots. Phospholipase Dζ1 can modulate the cellular polarity of auxin transport carriers (Korver et al., 2020). Another issue to be answered is the contribution of tissue-specific accumulation of pH-sensing phosphatidic acid to the halotropism (Li et al., 2019). Phophatidic acid is able to directly regulate the PINOID-dependent phosphorylation and activation of the PIN-FORMED2 auxin efflux transporter during salt stress (Wang P. et al., 2019).

Salt can induce remodeling of spatially restricted clathrin-independent endocytic pathways in *Arabidopsis* root (Baral et al., 2015). Endomembrane trafficking has a significant role in plant abiotic stresses (Wang et al., 2020). For example, Golgi-localized cation/proton exchangers regulate ionic homeostasis and skotomorphogenesis in *Arabidopsis* (Wang et al., 2018). Root bending is affected by auxin metabolism, protein phosphatase 2A and ABCB transporters activity (Han et al., 2017). Root apex proton fluxes show an important role in soil-stress acclimation (Siao et al., 2020).

Deolu-Ajayi et al. (2019) recently identified those genetic loci in natural accessions of *Arabidopsis thaliana* by genome-wide association study (GWAS) which could be involved in early salt stress responses of roots. Three candidate genes specific for halotropic movements were determined: *CHX13*, *WRKY25* and *DOB1*. *Arabidopsis thaliana* WRKY25 is coding a salt-inducible transcription factor which can mediate oxidative stress tolerance and senescence in a redox-dependent manner and also required for halotropic events (Jiang and Deyholos, 2009; Doll et al., 2020).

Proper K^+^ level has to be maintained during halotropic responses. AtCHX13, a cation proton exchanger is belonging to potassium transporter family. It is a plasma membrane K^+^ transporter (Zhao et al., 2008). Shabala (2017) supposed the probability of potassium to be involved in signaling as second messenger. CHX13 contributes the proper halotropic movements only under limiting potassium conditions, such as insufficient fertilizer application. It is important to note that maintaining plant intracellular K^+^ homeostasis during adverse saline conditions coexist with energy cost requirement (Rubio et al., 2020). Maybe AtDOB1 (Double Bending 1) could be specific for *Brassicaceae* and localized in cytosol with unexplored function (Lama et al., 2019), however, recent findings suggest that DOB1 might play a role in Na^+^/K^+^ accumulation during halotropism.

## Potential Secondary Messengers Involved in Halotropism

### Secondary Messengers

**Figure 2:**
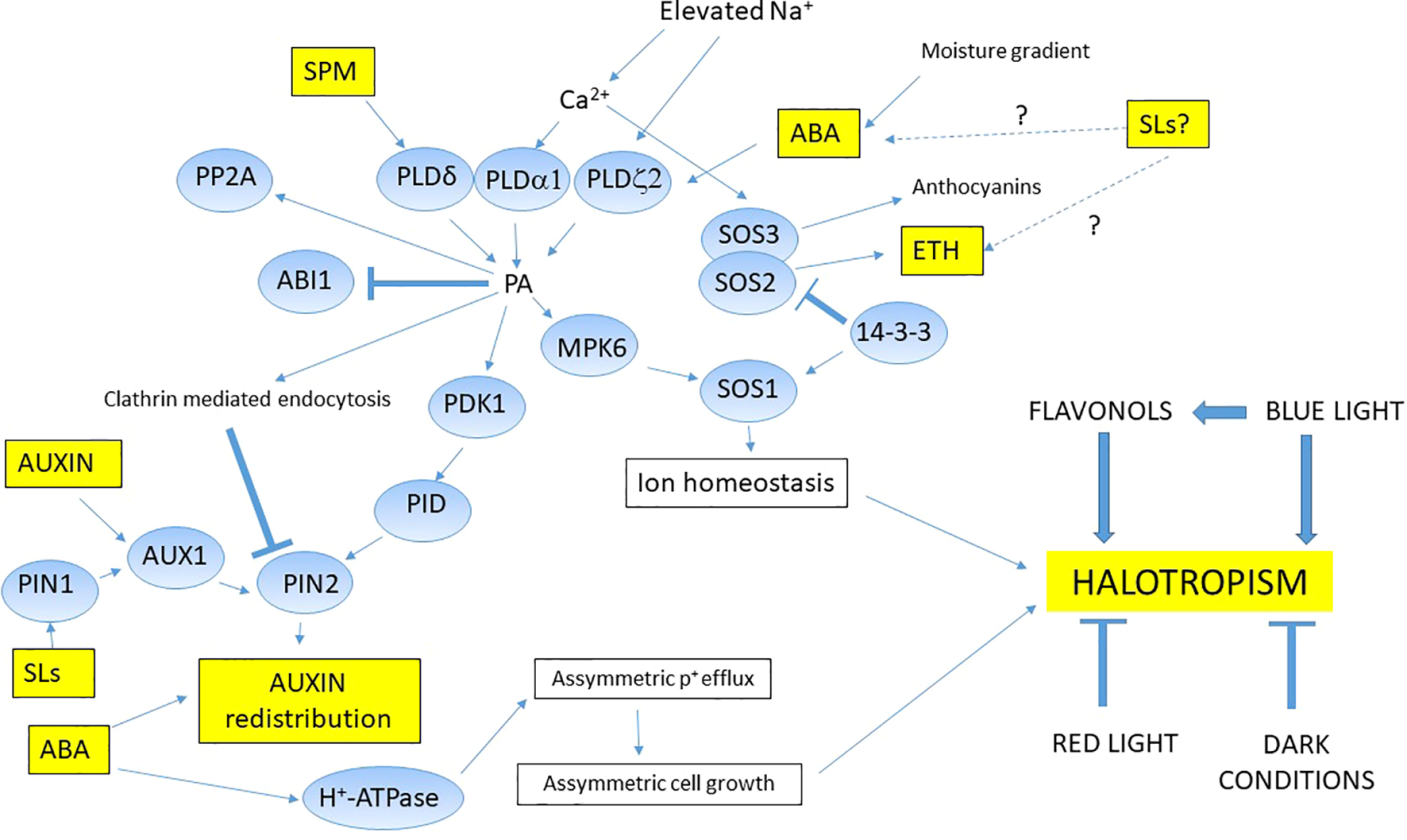
Proposed mechanism of halotropism in glycophytes. During halotropism, elevated Na^+^ triggers activation of phospholipid signal pathway by interacting PLDs through Ca^2+^ levels. The released phosphatidic acid (PA) could induce MAPKs which directly activate SOS1. Other factors such as 14-3-3 proteins also can activate SOS1 in order to fine tune and maintain the intracellular sodium content. PA can indirectly modulate the activity of PDK1 kinase (phosphoinositide-dependent kinase 1), the PID (PINOID) protein serine/threonine kinase, and the protein phosphatase 2A (PP2A) phosphatase complex, all three compounds involving in halotropism. PA also activates auxin transporter activities contributing to auxin redistribution and finally root bending. SLs can modulate the ABA and ethylene levels, indirectly affect the halotropic regulations. Auxin and ABA have crucial role in proper auxin redistribution during halotropism through PIN2. New evidences support that halotropism is differently affected by light conditions and secondary metabolites, however their exact mechanisms need to be deciphered. Question marks and dotted lines show us connections needed to be investigated in halotropism.

Flavonoids are good candidate to be positional signals in root growth responses as regulators in halotropism. The accumulation of flavonols induced by light could promote cell elongation and asymmetric growth in the root transition zone, so flavonols could serve as positional signals (Silva-Navas et al., 2016). Rough bluegrass (*Poa trivialis* L.), a flavonoid hyper-accumulating turfgrass species showed halotropic movements exposed to NaCl concentration gradients (Petrella et al., 2018). Light also can act as stress factor in the halotropic movements (Yokawa et al., 2014). Halotropism was enhanced in plants treated with blue light (BL) however red light or darkness did not induce halotropic growth. Flavonoids increased only in BL treated roots providing new evidence that BL and flavonoids are involved in regulating halotropism (**Figure 2**).

Some reactive oxygen and nitrogenous species are also suggested to be a part of signaling pathways of halotropic movements. Nitric oxide (NO), a gaseous molecule can be a good candidate for regulating multiple signal pathways during halotropism. It is accepted that NO has basic and essential role in root development and also under stress conditions (Corpas and Barroso, 2015). NO can interact with other signal compounds, like hydrogen peroxide or hydrogen sulfide, which are also able to produce endogenously (Corpas et al., 2019; Gohari et al., 2020; Singh et al., 2020). NO could mediate auxin accumulation and signaling in *Arabidopsis* and decrease the size of root meristem size during salt stress (Liu et al., 2015). Recently, Horváth et al. (2019) identified that two genes coding glutathione transferase enzymes, AtGSTF8 and AtGSTU19, GSTs from *Arabidopsis* can maintain the root redox homeostasis by affecting meristem size and salt stress sensitivity. NADPH oxidases generating superoxide anions in plant cells are center hubs during plant growth and signaling emphasizing the necessity to analyze its contribution to initiation or modulation of halotropism (Hu et al., 2020). Zwiewka et al. (2019) discovered the background of root adaptation to hydrogen peroxide-induced oxidative stress and the involvement of ARF-GEF BEN1- and cytoskeleton-mediated PIN2 trafficking in this process reflecting the possible implication of hydrogen peroxide in halotropism.

## Phytohormones Orchestrating Halotropic Events

### Auxin

This hormone is the most studied hormonal compound in halotropism. Auxin, a plant hormone is involved in a plethora of plant mechanisms not just in plant development but also in stress induced alterations (Korver et al., 2018). Auxin has critical role in the regulation of root cell elongation and tropic growth (Vanneste and Friml, 2009). Auxin transport regulation at posttranscriptional level by multiple hormonal pathways highlights the overlapping central role of auxin in development and stress processes (Semeradova et al., 2020). Auxin levels are different in plant species, e.g., low indole-acetic-acid (IAA) contents were measured in roots of some halophytes, e.g., *Prosopis strombulifera* (Llanes et al., 2019), indicating that different auxin levels might be responsible for different direction of halotropic bending. In *Limonium bicolor*, a recretohalophyte species, the root IAA levels enhanced under halotropic movements, however the exact mechanism needs to be deciphered. Detailed overview of factors which can include the bending model of halotropism is provided in the review of Han et al. (2017). Besides lipid signaling and protein phosphorylation cascades, auxin metabolism and transport also has a crucial part of halotropic signaling. The most studied factor involved in halotropic movements is the PIN2 auxin transporter internalization (**Figure 2**). Emenecker and Strader (2020) provided evidence about auxin-abscisic acid interactions suggesting a new regulation of halotropism. Also, it has been emerged an auxin-ethylene crosstalk at a systems level (Zemlyanskaya et al., 2018). Interestingly, newly discovered the antagonistic interactions between cytokinin signaling and auxin transport in shaping RSA for plant adaptation (Xiao and Zhang, 2020). Strengthening the importance of auxin and its metabolism in halotropism, further study of other plant species is needed.

### Abscisic Acid

Abscisic acid (ABA) is a sesquiterpene plant hormone involved in halotropism. It has many functions in plant development and abiotic stress tolerance as a general inhibitor of growth mechanisms, like primary root growth (Sun et al., 2018). ABA is a crucial in RSA modulation during environmental stress conditions (Harris, 2015), however evidence of ABA-mediated halotropic events in halophytes is missing. ABA can affect the lipid signal pathways activating PLD activities and adjusting auxin redistribution by PIN2 (**Figure 2**). However, PA binding to ABI1 (ABA Insensitive 1) can inhibit this protein phosphatase C (Ma et al., 2009). ABA regulates root elongation through the activities of auxin and ethylene in *Arabidopsis thaliana* and the biphasic root growth response to ABA require interaction with ethylene and auxin signaling pathways (Thole et al., 2014; Li et al., 2017). GWAS study revealed the importance of ABA and ethylene in the halotropic movements (Deolu-Ajayi et al., 2019). The halophytes specific mechanisms of ABA-regulated halotropism is unknown.

### Ethylene

Ethylene play a central role in an orchestrated process cooperating with other hormones in case of primary root growth and development (Qin et al., 2019). GWAS of natural *Arabidopsis thaliana* accessions studied by Deolu-Ajayi et al. (2019) revealed a cooperation between ABA and ethylene in regulating halotropism in roots to achieve a sustainable growth under adverse conditions. Also, PA can affect the ethylene response as binding to CTR1 (constitutive triple response 1), which step can further block the interaction between CTR1 and ETR1, ethylene receptor (Testerink et al., 2007).

### Strigolactones

Strigolactones (SLs) are new players in signaling pathways of plants (Al-Babili and Bouwmeester, 2015). Their participation was proved in root development and in abiotic stress related processes, or in interactions with the biotic soil microbiome (Kapulnik and Koltai, 2014; Koltai, 2014; Saeed et al., 2017; Jia et al., 2019a; Jia et al., 2019b). By using synthetic SL analog GR24 the SL-triggered alterations in RSA in *Arabidopsis thaliana* was stated (Ruyter-Spira et al., 2011). Wang J. Y. et al. (2019) investigated zaxinone induced growth and SL biosynthesis in rice. Metabolome analysis of SL-mutants and GR24 treated plants revealed that biosynthesis of flavonols are SL-dependent. In addition, flavonols function is also dependent from IAA and ABA, emerging new aspects for potential SL involvement in halotropism (**Figure 2**).

## Other Compounds With Potential Functions in Root Halotropism

Salicylic acid (SA) is a plant hormone belonging to plant phenolic secondary metabolites (Enyedi et al., 1992) could improve acclimation to salt stress by stimulating ABA accumulation and increasing Na^+^ content in leaves without any toxicity in tomato (Szepesi et al., 2009). SA induces different manner the ethylene and polyamine synthesis in proved evidence that SA differently impacts ethylene and polyamine synthesis in the glycophyte *Solanum lycopersicum* and the wild-related halophyte *Solanum chilense* exposed to mild salt stress (Gharbi et al., 2016). New evidence shows that this hormone can affect root meristem patterning *via* auxin distribution is a concentration dependent process (Pasternak et al., 2019). Also, SA can target protein phosphatase 2A to attenuate growth in plants (Tan et al., 2020), providing new potential signal component related in root tropisms.

By the newest technical and analytical approaches numerous new compounds are discovered nowadays suggesting their potential efficiency to modulate RSA. Apocarotenoids can be good candidate for halotropism induced components in plants, as they are involved in plant development and stress responses (Felemban et al., 2019). Anchorene is a carotenoid-derived regulatory metabolite which is required for anchor root formation in *Arabidopsis* (Jia et al., 2019). Also, β-cyclocitral is a newly discovered and characterized compound, which is a conserved root growth regulator, supposing its role in root tropisms (Dickinson et al., 2019). Yet, metabolites coordinating or regulating halotropic events have not been determined.

Polyamines (PAs) as essential polycations are regulators of a plethora of developmental and stress induced alterations (Bouchereau et al., 1999; Alcázar et al., 2010). Emerging interest has been added to study PAs in halophytes in salt tolerance (Bueno and Cordovilla, 2019), emphasizing the regulatory role of polyamines in abiotic stress as hub molecules (Sequera-Mutiozabal et al., 2017). Cooperating with other plant hormones such as cytokinin (Černý et al., 2013) and interacting with nitrogen in stress responses make them able to fine tune the proper C/N ratio in order to achieve the optimal conditions for growth or stress responses (Paschalidis et al., 2019). Moreover, PAs reprogramming oxidative and nitrosative status of salt exposed citrus plants could affect their redox status (Tanou et al., 2014). Newest findings suggest that PAs can adjust the quality control of post-transcriptional regulation (Poidevin et al., 2019). Some important N-containing metabolite like proline amino acid (Szepesi and Szollosi, 2018; Guan et al., 2020) or gamma-aminobutyric acid (GABA) as endproduct of PA catabolism could be involved in abiotic stress responses (Su et al., 2019). PA catabolism can synthesize secondary messengers like hydrogen peroxide or GABA (Wang W. et al., 2019), involved in sublethal and lethal salt stress (Takács et al., 2017). Recently, new results suggested that PAs can mediate halotropic events as tetraamine spermine in exogenously applied manner triggering a rapid intracellular phosphatidic acid response in *Arabidopsis* with PLDδ activation and ion flux stimulation (Zarza et al., 2019) (**Figure 2**). Halophytes can contain elevated polyamine levels dependent from plant species, age or organ, so polyamines are also promising targets for halotropic studies.

## Potential Application of Halotropism in Agriculture and Other Areas

Soil salinity of fields is often non-uniform. Xiong et al. (2020) suggested that the hormone signal transduction and the antioxidant pathway probably play important roles in inducing more salt-related genes and increasing resistance to non-uniform salt stress on both sides of the roots investigated in alfalfa. Recent studies show the needs to investigate a salt mixture or use non-homogenous salt concentrations in order to gain better understanding crop salt avoidance or foraging strategies. Also, Waidmann et al. (2020) reported that primary and lateral roots growth responses are differentially integrated root system growth. Primary and lateral roots perceive and integrate non-uniform salt conditions and may energy can allocate between these root types in case of glycophytes or halophytes (Ramezani et al., 2013; Wu et al., 2019). Also important issue to focus on invasive plants which has extraordinary surviving strategies in adverse conditions threatening the natural habitat (Bakacsy, 2019). Stress factors usually occur combined combination to each other affecting RSA (Osthoff et al., 2019; Sewelam et al., 2020). There is an increasing evidence about significance of root tropism in adjusting root system to changing conditions due to global climate change and inadequate agricultural procedures (Rozema and Schat, 2013; Gohari et al., 2020; Zhao et al., 2020). Halotropism can help roots to navigate and remodel their system architecture by cost effective energy supply in order to successfully survive during different salt conditions. Modulate RSA in order to adapting for rapidly and unexpectedly changing environment is inevitable process of plants (Waidmann et al., 2020). It should be keep in mind that these responses strongly depend of energy costs of plants during salt stress (Munns and Gilliham, 2015; Fricke, 2020; Munns et al., 2020). In the future, a big task to find good candidate plants differing salt avoidance and foraging mechanisms (e.g. euhalophytes, facultative halophytes and recretohalophytes) (Zarei et al., 2020). There is an increasing number of studies from investigation of halophyte-specific root growth (Yuan et al., 2018; Kiani-Pouya et al., 2020). To increase the halophyte feature our crop plants can provide an environmentally sustainable solution for increased crop yield in line with food demand (Liu et al., 2020). Genes which responsible for and metabolites involved in rapid and successful halotropic movements avoid salt stress can help us to integrate them or apply into salt sensitive crop plants increasing their tolerance against salt stress (Kosmacz et al., 2020). Also, transcriptomic analysis of monocot halophyte plants can reveal new data about their root tropism against salt (Ye et al., 2020). The next level can be to investigate these responses at system level (Zandalinas et al., 2020) monitoring the overlapping and cooperating proteins involved in root salt avoidance or foraging mechanism. One other possible mode of enhancing the salinity tolerance in our crop plants is the use of halotolerant microorganisms (Zhou et al., 2017; Etesami and Glick, 2020; Molina-Montenegro et al., 2020). There is some suitable experimental setup which can offer easily laboratorial assay to investigate the microbe related root tropic movements or deciphering some natural metabolites from plants which can mediate halotropic movements (Marik et al., 2019; Turbat et al., 2020). Since halophytes plants bear capability to survive adverse conditions even combined stress factors, like salt stress and heavy metal stress, they can efficiently use in phytoremediation purposes (Wani et al., 2020). Information about their root growth and their altered RSA can contribute to our knowledge (Yun et al., 2019) and help us to use marginal lands for more crop yield. Investigating root salt avoidance or salt directed movements is important in Earth and also in Space conditions to unravel the aspects and background mechanisms of sodium derived plant growth direction movements (Muthert et al., 2020).

## Author Contributions

The author confirms being the sole contributor of this work and has approved it for publication.

## Funding

This research was supported by NRDI (National Research, Development and Innovation) Office by Hungarian Ministry under the grant number FK129061 and the University of Szeged Open Access Fund (4786). 

## Conflict of Interest

The author declares that the research was conducted in the absence of any commercial or financial relationships that could be construed as a potential conflict of interest.
